# SsaV Interacts with SsaL to Control the Translocon-to-Effector Switch in the *Salmonella* SPI-2 Type Three Secretion System

**DOI:** 10.1128/mBio.01149-18

**Published:** 2018-10-02

**Authors:** Xiu-Jun Yu, Grzegorz J. Grabe, Mei Liu, Luís Jaime Mota, David W. Holden

**Affiliations:** a MRC Centre for Molecular Bacteriology and Infection, Imperial College London, London, United Kingdom; b UCIBIO-REQUIMTE, Departamento de Ciências da Vida, Faculdade de Ciências e Tecnologia, Universidade Nova de Lisboa (FCT NOVA), Caparica, Portugal; University of Utah; Washington University School of Medicine

**Keywords:** *Salmonella*, effector, secretion switch, translocon, type III secretion

## Abstract

Nonflagellar type III secretion systems (nf T3SSs) form a cell surface needle-like structure and an associated translocon that deliver bacterial effector proteins into eukaryotic host cells. This involves a tightly regulated hierarchy of protein secretion. A switch involving SctP and SctU stops secretion of the needle protein. The gatekeeper protein SctW is required for secretion of translocon proteins and controls a second switch to start effector secretion. Salmonella enterica serovar Typhimurium encodes two T3SSs in *Salmonella* pathogenicity island 1 (SPI-1) and SPI-2. The acidic vacuole containing intracellular bacteria stimulates assembly of the SPI-2 T3SS and its translocon. Sensing the nearly neutral host cytosolic pH is required for effector translocation. Here, we investigated the involvement of SPI-2-encoded proteins SsaP (SctP), SsaU (SctU), SsaV (SctV), and SsaL (SctW) in regulation of secretion. We found that SsaP and SsaU are involved in the first but not the second secretion switch. A random-mutagenesis screen identified amino acids of SsaV that regulate translocon and effector secretion. Single substitutions in subdomain 4 of SsaV or InvA (SPI-1-encoded SctV) phenocopied mutations of their corresponding gatekeepers with respect to translocon and effector protein secretion and host cell interactions. SsaL interacted with SsaV in bacteria exposed to low ambient pH but not after the pH was raised to 7.2. We propose that SsaP and SsaU enable the apparatus to become competent for a secretion switch and facilitate the SsaL-SsaV interaction. This mediates secretion of translocon proteins until neutral pH is sensed, which causes their dissociation, resulting in arrest of translocon secretion and derepression of effector translocation.

## Abstract (precis)

*Salmonella* Typhimurium is an intracellular pathogen that uses the SPI-2 type III secretion system to deliver virulence proteins across the vacuole membrane surrounding intracellular bacteria. This involves a tightly regulated hierarchy of protein secretion controlled by two molecular switches. We found that SPI-2-encoded proteins SsaP and SsaU are involved in the first but not the second secretion switch. We identify key amino acids of the inner membrane protein SsaV that are required to interact with the so-called gatekeeper protein SsaL and show that the dissociation of SsaV-SsaL causes the second switch, leading to delivery of effector proteins. Our results provide insights into the molecular events controlling virulence-associated type III secretion and suggest a broader model describing how the process is regulated.

## INTRODUCTION

**FIG 1:**
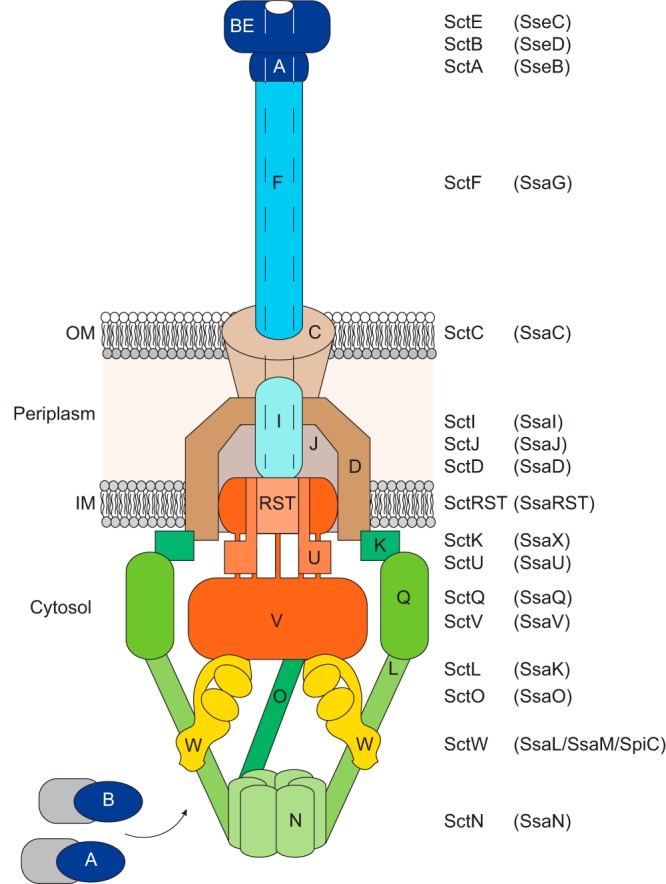
Schematic drawing of T3SS. The unified nomenclature Sct (secretion and cellular translocation [1]) names are used to label the components of T3SS, and the corresponding components of SPI-2 T3SS are indicated in parentheses. The cartoon represents the secretion state of translocon proteins of SPI-2 T3SS. Cytoplasmic sorting platforms are shaded in green, export gates in red, gatekeepers in yellow, basal bodies in brown, needles and inner rods in light blue, and translocon proteins in blue. IM, inner membrane; OM, outer membrane.

Nonflagellar type III secretion systems (nf T3SSs) are used by many Gram-negative bacterial pathogens to deliver virulence (effector) proteins directly from the bacterial cytosol into host cells through a hollow needle-like filament that extends from the bacterial cell surface and an associated translocon pore that is formed in the host cell membrane (1–5). Effectors can block phagocytosis or promote bacterial invasion into nonphagocytic cells, modify membrane trafficking, and interfere with both innate and adaptive immune functions (6, 7). These activities enable bacteria to survive and replicate, thereby causing disease.

nf T3SSs are composed of different subsets of proteins (Fig. 1), with various degrees of similarity between different bacteria. They comprise a conserved core of five proteins (SctRSTUV) (8), spanning the bacterial inner membrane to form an export gate that is connected to two other inner membrane proteins (SctDJ) and an outer membrane ring (the secretin [SctC]). SctV is a multi-membrane-spanning protein with a large C-terminal cytoplasmic region and is an essential component of the export gate (9–11). This gate interacts on its cytoplasmic face with a sorting platform consisting of SctO, the C-ring protein (SctQ), and an ATPase (SctN) and its regulators and enables sequential loading of substrates prior to their secretion (12–15). A cytoplasmic “gatekeeper” complex (including SctW) is required for translocon protein secretion and to prevent premature secretion of effectors (16–18).

Effector translocation is a tightly regulated process, characterized by the ordered secretion of different proteins. After the secretion apparatus has assembled, subunits of the surface filament (SctF) are secreted, along with an inner rod protein (SctI) and a “ruler” protein (SctP) that regulates needle length (19–21). When the filament reaches a defined length, a secretion switch occurs in which filament subunit secretion is halted and translocon proteins are secreted. In some systems, these include subunits of a tip complex (22–24) or a long sheath-like filament (25, 26), and all systems secrete proteins that form a translocon pore in the target cell membrane that is required for effector translocation (22, 26, 27). Once the pore has formed, a second switch occurs, characterized by cessation of translocon protein secretion and derepression of effector translocation.

In addition to being secreted, SctF and SctI are essential for secretion of SctP, translocon and effector proteins (19), whereas SctP regulates needle length and is not required for secretion of SctF and SctI (20, 21, 28, 29). The first secretion switch is controlled by SctP, together with SctI and SctU. SctU has a conserved NPTH motif in its cytoplasmic domain, which is a site for autocleavage (30). Prevention of cleavage of SpaS, the SctU of the T3SS encoded by *Salmonella* pathogenicity island 1 (SPI-1) (by replacing asparagine [N] with alanine [A] in the NPTH sequence), abolished secretion of translocon and effector proteins but not inner rod and ruler proteins (28), whereas the same mutation in YscU, the SctU protein of Yersinia enterocolitica, drastically reduced secretion of translocon but not effector proteins (31, 32).

The regulatory effects of SctP in secretion switches also differ among T3SSs. Deletion of *invJ*, the gene encoding the SctP ruler protein of the SPI-1 T3SS, abolished secretion of translocon and effector proteins but not the inner rod protein (28). On the other hand, deletion of *escP*, the gene encoding the SctP protein of enteropathogenic Escherichia coli (EPEC), resulted in enhanced secretion of effectors and decreased secretion of translocon proteins (33)—a secretion profile similar to that of gatekeeper mutants (*sepL* or *sepD*) of EPEC (17).

The SctW gatekeeper proteins include EPEC SepL, *Salmonella* SPI-1 InvE, *Salmonella* SPI-2 SsaL, *Shigella* MxiC, *Yersinia* YopN/TyeA, and Pseudomonas aeruginosa PopN/Pcr1. Deletion of *sepL*, *invE*, *ssaL,* or *mxiC* causes deregulated secretion of effectors and impairs or blocks secretion of translocon proteins (16–18, 34), demonstrating that these proteins control the second secretion switch. Although deletion of *yopN* or *tyeA* or of *popN* or *pcr1* resulted in constitutive secretion of both translocon and effector proteins, these proteins are required for proper translocation of effectors into host cells (35–38). Upon the second switch, MxiC, YopN, and PopN are all secreted (34–36, 39) whereas SepL, InvE, and SsaL are not (16–18). A point mutation of YopN (YopN^F234S^), which is not secreted, blocked secretion of translocon and effector proteins (40), indicating that the gatekeeper might interact with a component(s) of the secretion system. Indeed, it was shown that Pcr1 interacts with PcrD, the inner membrane SctV protein of P. aeruginosa (41); that MxiC interacts with MxiA, the SctV protein of *Shigella* (42); and that SepL interacts with EscV, the SctV protein of EPEC (43, 44). A point mutant of PcrD (Q626R) that no longer interacts with Pcr1 led to deregulation of effector secretion, a phenotype that appears to mimic that of the *pcr1* mutant (41). However, the effect of PcrD^Q626R^ on secretion of translocon proteins was not described. A point mutant of MxiA (MxiA^I674V^) that failed to interact with MxiC in a bacterial two-hybrid assay had only a minor effect on regulation of secretion of translocon and effector proteins (42).

Salmonella enterica is an invasive intracellular pathogen that encodes two T3SSs. The SPI-1 T3SS is activated in extracellular bacteria and mediates invasion of mammalian cells. The SPI-2 T3SS is activated by intravacuolar bacteria, and its effectors interfere with many processes, collectively enabling systemic growth of bacteria in host tissues (7). InvE functions as a gatekeeper for the SPI-1 T3SS (16), while a complex of SsaL, SsaM, and SpiC represents the SPI-2 gatekeeper complex that regulates the switch from translocon to effector protein secretion (18). None of the *Salmonella* gatekeeper proteins appear to be secreted (16, 18). Assembly of the SPI-2 T3SS and secretion of its translocon proteins are activated by nutritional deprivation and low pH (approximately pH 5.0) of the lumen of intracellular vacuoles (45–47). Once the translocon has formed in the vacuole membrane, the nearly neutral pH of the host cell cytoplasm is detected, leading to disassembly of the SsaL/SsaM/SpiC gatekeeper complex. This results in suppression of translocon protein secretion and translocation of effectors (18). How SsaL functions as the gatekeeper is unknown, and the relationship between the gatekeeper and the secretion apparatus in regulating translocon and effector secretion remains poorly understood.

To gain more insight into the mechanism by which the SPI-2 T3SS gatekeeper regulates secretion, we investigated the role of SsaP, SsaU, and SsaV (corresponding to SctP, SctU, and SctV, respectively) in secretion of translocon and effector proteins. We found that SsaP and autocleavage of SsaU are essential for the first secretion switch. We identified a single amino acid residue in the conserved sequence of subdomain 4 of SsaV that is essential for stable interaction between SsaV and SsaL in bacterial cells exposed to pH 5.0. In wild-type (wt) cells, the interaction between SsaV and SsaL was lost after a change in ambient pH to pH 7.2. These results indicate that the second secretion switch dissociates the gatekeeper complex from the secretion apparatus, leading to derepression of effector translocation.

## RESULTS

### Effect of SsaP and autocleavage of SsaU on the secretion switch.

**FIG 2:**
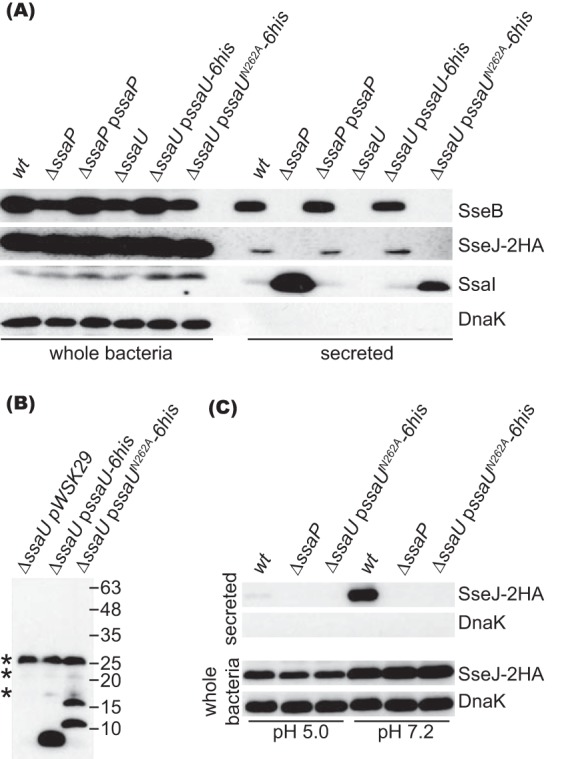
SsaP and autocleavage of SsaU are essential for secretion of SseB and SseJ-2HA. (A) Levels of SseB, SseJ-2HA, and SsaI in whole bacteria and secreted fractions of different strains grown in magnesium minimal medium-morpholineethanesulfonic acid (MgM-MES) at pH 5.0. (B) The cleavage pattern of SsaU-6his and SsaU^N262A^-6his. Whole-bacterium lysates were separated by SDS-PAGE, transferred to a PVDF membrane, and probed with an anti-His antibody. Asterisks (*) indicate nonspecific bands. (C) Bacterial strains were grown in MgM-MES at pH 5.0 for 4 h, which was then changed to fresh MgM-MES at pH 5.0 or 7.2 as indicated, and the strains were then incubated for 1.5 h before analysis.

To investigate the requirement of SsaP in secretion switches, we created an *ssaP* deletion mutant (Δ*ssaP*) and carried out a secretion assay after growing bacterial strains in MgM-MES at pH 5.0 to mimic the vacuole lumen. Under these conditions, wt bacteria secreted the translocon protein SseB but suppressed secretion of an epitope-tagged effector, SseJ-2HA, and of the inner rod protein SsaI (Fig. 2A). The Δ*ssaP* mutant hypersecreted SsaI but failed to secrete SseB or SseJ-2HA (Fig. 2A). Introducing a functional allele of *ssaP* on a plasmid (p*ssaP*) into the Δ*ssaP* mutant restored secretion of SsaI, SseB, and SseJ to the levels of the wt strain. Therefore, SsaP is required for secretion of translocon and effector proteins and its absence deregulates secretion of SsaI.

Like other SctU proteins, SsaU has the conserved NPTH sequence (residues 262 to 265) and the uncleaved full-length protein has a predicted mass of 40 kDa. We tagged *ssaU* with a 6-histidine sequence at its C terminus and expressed it from plasmid p*ssaU-6his*. Immunoblotting failed to detect a protein of 40 kDa but indicated that SsaU, like its homologues, was processed to produce a C-terminal fragment smaller than 10 kDa. When N262 was replaced with alanine (A), two new peptides were produced, both larger than 10 kDa (Fig. 2B), indicating that alternative cleavage sites are generated in SsaU^N262A^, as previously observed with YscU^N263A^ (31). This result indicates that SsaU is efficiently cleaved between N262 and P263 once it is synthesized.

We tested the requirement for correct cleavage of SsaU in secretion by growing bacterial strains in MgM-MES at pH 5.0. As expected, deletion of *ssaU* abolished secretion of SsaI, SseB, and SseJ (Fig. 2A). The secretion defect of the Δ*ssaU* mutant was rescued to wt levels by introducing plasmid p*ssaU-6his*. However, expression of SsaU^N262A^ in the Δ*ssaU* mutant blocked secretion of SseB and SseJ, despite having a functional secretion system, as shown by enhanced secretion of SsaI (Fig. 2A). This shows that cleavage of SsaU between N262 and P263 is required for secretion of translocon and effector proteins and for regulated secretion of SsaI. Since secretion of effectors by the SPI-2 T3SS is suppressed when bacteria are grown at pH 5.0, we tested the ability of the Δ*ssaP* mutant or of the Δ*ssaU* mutant expressing SsaU^N262A^ to secrete SseJ after increasing the ambient pH from 5.0 to 7.2. Secretion of SseJ-2HA was derepressed in wt bacteria but prevented in the Δ*ssaP* mutant and the Δ*ssaU* mutant expressing SsaU^N262A^ (Fig. 2C). Therefore, SsaP and specific cleavage of SsaU are required for controlling secretion from SsaI to translocon and effector proteins.

### The C-terminal nine amino acids of SsaV are required for efficient control of translocon and effector protein secretion.

**FIG 3:**
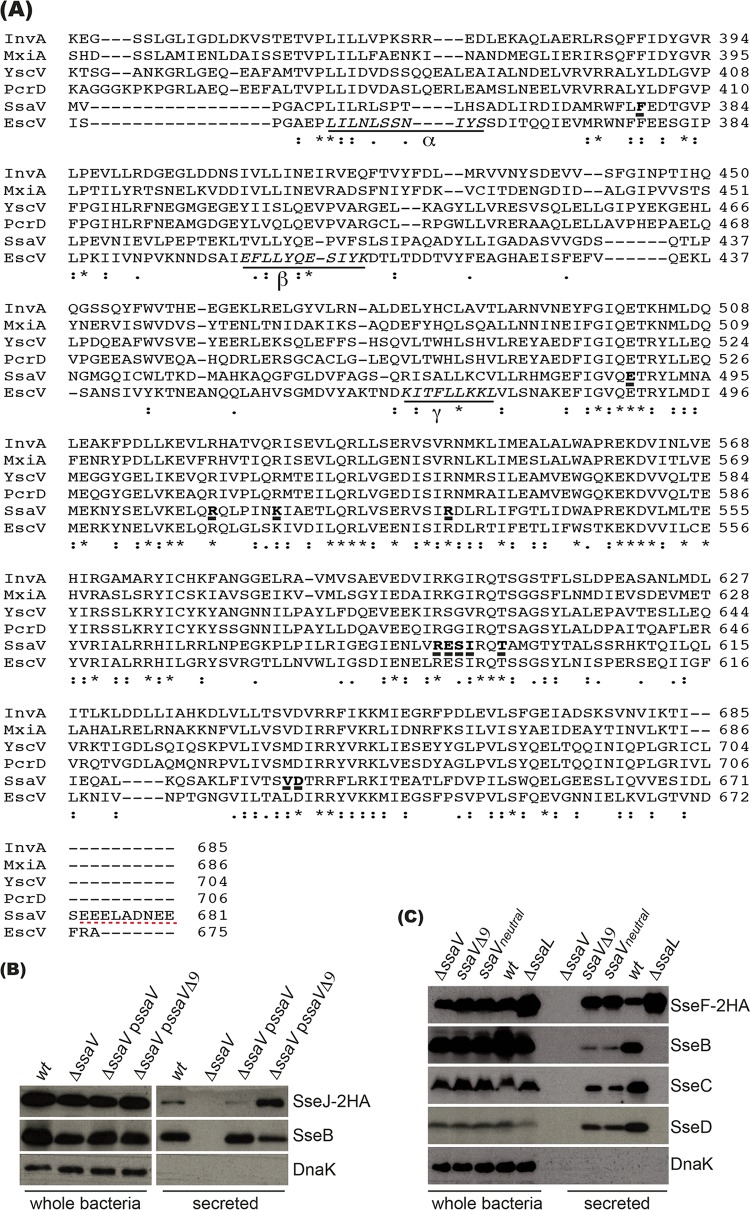
Contribution of the C-terminal nine amino acids of SsaV to SPI-2 type III secretion. (A) Alignment of the C-terminal region of SsaV and its homologues. SsaV and its homologues were aligned using Clustal Omega. The last nine residues of SsaV are underlined with red dashed lines; the mutations of SsaV shown in Fig. 4 are indicated with bold and with a thick underline. The peptide sequences of EscV interacting with SepL by peptide array assay (43) are indicated with α, β, and γ. GenBank accession numbers for proteins are as follows: for InvA, NP_461817.1 (*S.* Typhimurium LT2); for MxiA, YP_009062504.1 (S. flexneri 5a M90T); for YscV, AAD16818.1 (Y. enterocolitica W22703); for PcrD, AAG05092.1 (P. aeruginosa PA01); for SsaV, NP_460379.1 (*S.* Typhimurium LT2); for EscV, AAK26714.1 (EPEC E234869). (B) Secretion analysis of Δ*ssaV* mutant carrying plasmid p*ssaV* or p*ssaVΔ9*. Bacterial strains expressing SseJ-2HA from the chromosome were grown in MgM-MES at pH 5.0 for 6 h, and whole-bacterium lysates and secreted fractions were subjected to immunoblot analysis. Intrabacterial protein DnaK was used as a control. (C) Chromosomally expressed SsaVΔ9 and SsaV_neutral_ strains partially mimic the Δ*ssaL* mutant. Data represent results of secretion analysis of the indicated strains, all carrying plasmid p*sseF-2HA*.

Alignment of SsaV and its homologues revealed that SsaV is unique in having an extended C terminus containing six negatively charged residues in the last nine amino acids, namely, EEELADNEE (Fig. 3A). To test the possible involvement of this sequence in secretion, we constructed plasmid p*ssaV* to express wt SsaV and plasmid p*ssaVΔ_9_* to express SsaV lacking the last nine residues (SsaVΔ_9_). The plasmids were introduced into a Δ*ssaV*::*aphT* null mutant expressing SseJ-2HA from the chromosome, and secretion of SseJ-2HA and SseB was examined by growing bacteria in MgM-MES at pH 5.0. Expression of SsaV in the Δ*ssaV*::*aphT* mutant restored secretion of SseB and SseJ-2HA to the levels of the wt strain, whereas expression of SsaVΔ_9_ in the Δ*ssaV*::*aphT* mutant led to increased secretion of SseJ-2HA and decreased secretion of SseB (Fig. 3B).

To test if this altered secretion profile was due to overexpression of SsaVΔ_9_, the DNA sequence encoding the last nine residues of SsaV was deleted from chromosomal DNA to create an *ssaVΔ_9_* strain, which was then transformed with p*sseF-2HA*. Compared to the wt strain results, expression of chromosomal SsaVΔ_9_ resulted in increased secretion of SseF-2HA and decreased secretion of translocon proteins SseB, SseC, and SseD (Fig. 3C). Furthermore, replacing the chromosomal coding sequence for the acidic amino acids in EEELADNEE with that of their corresponding amides (*ssaV_neutral_*) also led to increased secretion of SseF and decreased secretion of SseB, SseC, and SseD (Fig. 3C). Together, the results demonstrate that the six C-terminal negatively charged amino acids of SsaV are involved in regulating the secretion of effector and translocon proteins.

### Mutations in SsaV that phenocopy gatekeeper mutants.

Strains expressing SsaVΔ_9_ or SsaV_neutral_ partially mimicked a gatekeeper mutant for secretion of effectors and translocon proteins (Fig. 3C), suggesting that SsaV is also involved in the switch from translocon to effector secretion. Therefore, we conducted a screen to search for a mutation(s) of SsaV that completely phenocopied the gatekeeper mutants. To screen for such a mutation(s), p*ssaV* was randomly mutagenized in E. coli by error-prone PCR. Plasmids from 6,000 transformants were then isolated from E. coli, pooled, and transformed into the Δ*ssaV*::*aphT* mutant expressing SseJ-2HA from the chromosome.

**FIG 4:**
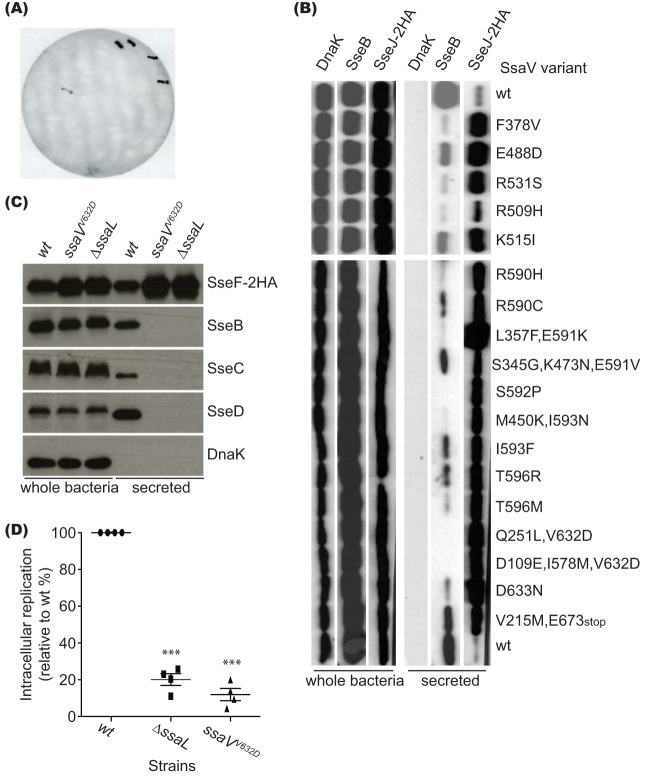
Screening and verification of SsaV variants phenocopying the secretion profile of the Δ*ssaL* mutant. (A) Example of dot immunoblotting of approximately 100 colonies to screen for increased secretion of SseJ-2HA. (B) Western blotting of SsaV variants leading to increased secretion of SseJ-2HA and decreased or undetectable secretion of SseB. The Δ*ssaV* mutant expressing SseJ-2HA from the chromosome and carrying a plasmid expressing wt SsaV or mutated versions was subjected to secretion assay. (C) The *ssaV^V632D^* strain phenocopies the Δ*ssaL* mutant. Data represent results of secretion analysis of the indicated strains, all carrying plasmid p*sseF-2HA.* (D) Replication assay in RAW264.7 macrophages. The intracellular replication (fold increase at 16 h/2 h) was normalized to that of the wt. ***, *P*  < 0.001.

We screened 18,500 *Salmonella* transformants for increased secretion of SseJ-2HA on solid MgM-MES pH 5.0 by dot blotting. A representative result is shown in Fig. 4A. A total of 469 clones that displayed enhanced secretion of SseJ-2HA were examined further by Western blotting to check secretion of SseJ and SseB. Of these, 216 transformants showed increased secretion of SseJ and decreased or no secretion of SseB. The plasmids from all 216 transformants were isolated and sequenced to define the mutation(s) in SsaV. These comprised single to multiple substitutions, repetitions of the same mutation(s), and frameshifts causing truncations lacking the C-terminal negatively charged amino acids. We focused on 18 mutants having a small number of substitutions and relatively strong phenotypic effects. In addition to mutation of E673stop (which expresses the SsaVΔ_9_ variant), mutations were localized in two regions: residues 378 to 531 (F378, E488, R509, K515, and R531) and residues 590 to 633 (R590, E591, S592, I593, T596, V632, and D633). Increased secretion of SseJ was confirmed in all 18 mutants tested (Fig. 4B). Furthermore, secretion of SseB was decreased by single mutation of F378, E488, R509, K515, R531, R590, I593, T596, and D633 or by mutations of E591 with others, and the S592P substitution and mutations containing V632D completely abolished secretion of SseB (Fig. 4B).

To test if a mutant with a single V632D substitution would completely mimic a gatekeeper mutant, we changed the chromosomal coding sequence for SsaV V632 to D in the wt strain to create strain *ssaV^V632D^*. The *ssaV^V632D^* strain failed to secrete translocon proteins SseB, SseC, and SseD but secreted more SseF than the wt strain, and its secretion profile was indistinguishable from that of the *ssaL* mutant (Fig. 4C). Like the *ssaL* mutant, the *ssaV^V632D^* strain also had a replication defect in mouse RAW macrophages (Fig. 4D). These results demonstrate that the region between F378 and D633 of SsaV is involved in the translocon-to-effector switch of the SPI-2 T3S and that the *ssaV^V632D^* and *ssaV^S592P^* mutants phenocopy the secretion profile of gatekeeper mutants.

### An *invA^V648D^* mutant mimics gatekeeper mutant Δ*invE* of the SPI-1 T3SS.

**FIG 5:**
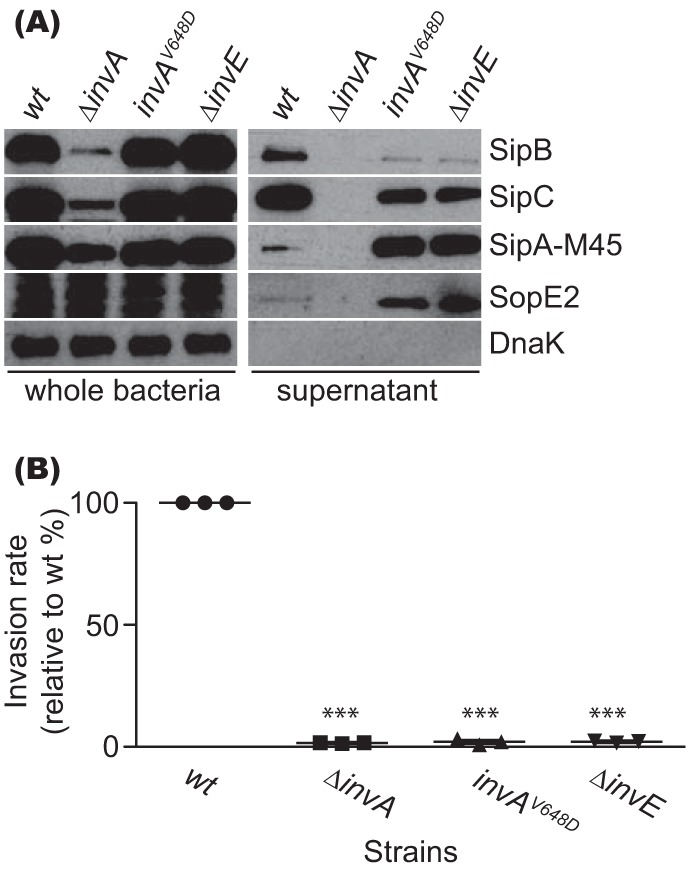
An InvA^V648D^ strain phenocopies the Δ*invE* mutant strain. (A) Secretion assay. Bacteria were grown to the exponential phase, and protein samples were prepared from whole bacteria and secreted fractions for immunoblotting. (B) Invasion assay. HeLa cells were infected with exponential-phase bacteria for 2 h to calculate the invasion rate. The invasion rate was normalized to that of the wt. ***, *P*  < 0.001.

Since the *ssaV^V632D^* mutant mimics the *ssaL* mutant and since both valine and its flanking amino acids (SVD) (residues 631 to 633) are conserved in the SPI-1 InvA homologue (residues 647 to 649; Fig. 3A), we asked if a corresponding mutation of InvA would mimic the phenotype of the SPI-1 gatekeeper *invE* mutant. For this purpose, we made a Δ*invE* mutant and also made an *invA^V648D^* mutant in which the coding sequence of V648 of InvA was replaced with the codon for D on the chromosome. Plasmid pM1301, which expresses M45-tagged effector SipA under the control of a SPI-1 promoter, was transformed into bacterial strains for secretion analysis. As shown in Fig. 5A, the *invA^V648D^* mutant had a secretion profile very similar to that of the Δ*invE* mutant; they both secreted lower amounts of translocon proteins SipB and SipC than the wt strain and far greater amounts of effectors SipA-M45 (plasmid expressed) and SopE2 (chromosome expressed). Consistent with the secretion profile, both the *invA^V648D^* mutant and the Δ*invE* mutant were defective for invasion of epithelial cells (Fig. 5B). Therefore, the *invA^V648D^* mutant phenocopies the Δ*invE* mutant with respect to secretion and host cell invasion, indicating a common function of InvA and SsaV in cooperating with their respective gatekeepers to control the switch from translocon protein secretion to effector protein secretion.

### SsaV interacts with SsaL.

**FIG 6:**
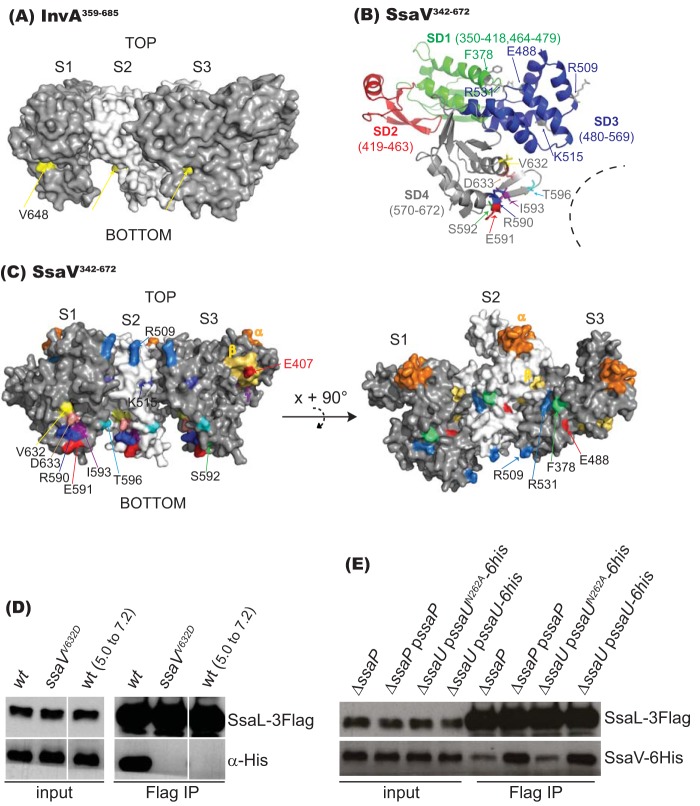
Distribution of mutations of SsaV and interaction between SsaL and SsaV. (A) View from lumen of InvA trimer. S1, S2, and S3 represent three subunits of InvA. V648 is indicated with yellow arrows. (B) Location of residues of SsaV important for regulation of secretion of translocon and effector proteins. SD1, SD2, SD3, and SD4 indicate the four subdomains of the cytosolic region of SsaV modeled using the structure of MxiA. The dashed line indicates the inner ring surface. The residues and their corresponding subdomain are labeled with same color. (C) Lumenal view of SsaV and its mutations. S1, S2, and S3 represent three subunits of SsaV. The α, β, and γ patches of SsaV (predicted from EscV) are indicated. E407 (red font) of the β patch is predicted to form an intersubunit salt bridge with R531. Residues labeled with black font represent mutations of SsaV revealed in this work. (D) Interaction between SsaL and SsaV. Bacterial strains were grown under the following conditions: wt and *ssaV^V632D^* in MgM-MES at pH 5.0 for 6 h; wt (5.0 to 7.2) in MgM-MES at pH 5.0 for 4 h, with the medium changed to MgM-MES at pH 7.2 for 2 h. SsaL-3Flag was immunoprecipitated with anti-Flag M2 affinity agarose gel and detected with anti-Flag antibody. Anti-His antibody was used to detect SsaV-6his [lanes wt and wt (5.0 to 7.2)] and SsaV^V632D^-6his (lane *ssaV^V632D^*). (E) Effect of SsaP and autocleavage of SsaU on SsaL-SsaV interaction. Bacterial strains expressing SsaL-3Flag and SsaV-6His from chromosomal DNA were grown for 6 h in MgM-MES at pH 5.0. SsaV-6His coimmunoprecipitated (IP) with SsaL-3Flag was analyzed by immunoblotting.

The crystal structure of the cytosolic region of InvA (InvA^357-685^) contains four subdomains (SD1 to SD4) (10), an arrangement that is similar to the structure of the cytosolic region of MxiA (MxiA^318–686^) (11). Purified MxiA^318–686^ forms a nonameric ring in which SD2 faces the outside of the ring; parts of SD3 face the inner pore; and SD4 is located at the lower, inner part of the structure (11). This is consistent with an *in situ* cryoelectron tomography study on InvA, whose cytosolic region also forms a nonameric ring (13). The V648 of InvA is located within SD4 on the lower inner surface of each InvA subunit (Fig. 6A). Using the crystal structure of MxiA^318-686^ as a model, we mapped the mutations of SsaV shown in Fig. 4B as follows: F378V is in SD1 (residues 350 to 418 and 464 to 479); E488D, R509H, K515I, and R531S are in SD3 (residues 480 to 569); and R590H/C, E591K/V, S592P, I593F/N, T596R/M, V632D, and D633N are in SD4 (residues 570 to 672) (Fig. 6B). Residues E488 and R531 are predicted to form intersubunit salt bridges with R534 and E407, respectively (corresponding to E502-K548 and R545-E418 of MxiA) (11). Substitution F378V might have an effect on the R531-E407 salt bridge, as F378 is next to R531 on the surface map (Fig. 6C). E407 of SsaV is part of a region which in EPEC EscV is referred to as the β patch, implicated in binding of EscV to the SepL gatekeeper protein (43). R590, E591, S592, I593, T596, V632, and D633 are located within SD4 on the lower inner surface of each SsaV subunit, which forms a cleft between subunits (Fig. 6B and C). This suggests that the clefts formed among the C termini of SsaV subunits might accommodate the gatekeeper complex to control secretion. To investigate interactions between SsaV and SsaL and the importance of V632, we tagged endogenous SsaL with a 3-Flag epitope and SsaV or SsaV^V632D^ with 6×His epitopes and subjected them to a coimmunoprecipitation assay. SsaV-6×His but not SsaV^V632D^-6×His was reproducibly coimmunoprecipitated with SsaL-3Flag (Fig. 6D).

We have shown that the membrane-associated gatekeeper SsaL/SsaM/SpiC complex dissociates upon sensing of neutral pH, thereby derepressing effector translocation (18). The interaction between SsaV and SsaL and the similar secretion profiles of the *ssaV^V632D^* and *ssaL* mutant strains suggested that neutral pH might result in dissociation of these proteins. To test if the SsaV-SsaL interaction responds to pH change, bacterial strains expressing SsaV-6×His and SsaL-3Flag were grown for 4 h in MgM-MES at pH 5.0 and then incubated in MgM-MES at pH 7.2 for another 2 h. Bacterial cells were lysed, and SsaL-3Flag was immunoprecipitated and analyzed by immunoblotting. The interaction between SsaV-6×His and SsaL-3Flag was lost following an increase in ambient pH to 7.2 (Fig. 6D). Taken together, these results indicate that SsaL mediates secretion of translocon proteins by interacting with SsaV and that their dissociation is a physiological consequence of sensing neutral pH, which results in arrest of translocon protein secretion and derepression of effector secretion.

Finally, we tested if SsaP and normally cleaved SsaU contribute to the SsaV-SsaL interaction. To do this, we made Δ*ssaP* or Δ*ssaU* mutations in the strain expressing SsaV-6×His and SsaL-3Flag from their endogenous loci and then transferred plasmids expressing SsaP or SsaU^N262A^ into the mutants to test the interaction between SsaV and SsaL. There was less SsaV-6×His coimmunoprecipitated with SsaL-3Flag in the absence of SsaP (the level decreased by approximately 43.65 ± 4.11%) or in the absence of normally cleaved SsaU (the level decreased by approximately 46.18 ± 4.46%) (Fig. 6E), demonstrating that SsaP and normally cleaved SsaU are required for an efficient interaction between SsaV and SsaL.

## DISCUSSION

The mechanism by which T3SSs control the second secretion switch enabling effector translocation following assembly of the translocon pore is poorly understood. In the case of the *Salmonella* SPI-2 T3SS, effector secretion is repressed by low pH of the vacuole lumen and is activated only after assembly of the translocon pore. An unknown mechanism enables the nearly neutral cytoplasmic pH of the host cell to be sensed, leading to disassembly of the gatekeeper complex (SsaL/SpiC/SsaM) and effector translocation (18). This process can be simulated *in vitro* by exposing bacteria to pH 5.0 to induce assembly of the secretion system and secretion of translocon proteins, then raising the ambient pH to 7.2 to suppress translocon secretion and derepress effector secretion. We exploited this to assess the influence of three proteins (SsaU, SsaP, and SsaV) on the second secretion switch.

We were unable to detect full-length SsaU, suggesting that it is rapidly cleaved after translation, probably by autoproteolysis (30). Furthermore, a mutant version lacking a critical N262 residue led to aberrant cleavage and prevented secretion of SseB and SseJ. Therefore, highly specific cleavage of SsaU is essential for both translocon and effector protein secretion. The complete absence of SsaP also led to undetectable secretion of both SseB and SseJ, arguing against a significant contribution of these proteins in the translocon-to-effector switch. This is in contrast to the situation in other T3SSs, where EscP and specific cleavage of YscU are required for the switch from secretion of needle/inner rod proteins to translocon proteins (but not effectors) and where EscP is involved in the second switch (31–33, 48).

SctV is a large multi-membrane-spanning inner membrane protein that is essential for the assembly of the T3SS (11). SctV proteins are predicted to have 8 transmembrane helices and a large C-terminal cytoplasmic region composed of four subdomains (SD1 to SD4) (9–11). The cytoplasmic domains of SctV proteins MxiA and InvA assemble into nonameric rings (11, 13), suggesting that they are likely to be a general feature of all T3SSs. The SPI-2-encoded SctV protein SsaV has a unique 9-amino-acid C-terminal tail, within which 6 residues are negatively charged (Fig. 3). Mutation of these partially mimicked the secretion profile of the gatekeeper *ssaL* mutant in that it enhanced secretion of effectors and decreased secretion of translocon proteins (Fig. 3), suggesting that SsaV is intimately involved in the second secretion switch. To search for other regions of the protein that might be involved in regulating effector secretion, a more comprehensive mutagenesis of SsaV was undertaken. This led to the identification of several single, double, and triple substitutions in the C-terminal region that enhanced effector secretion and decreased secretion of translocon proteins. Of these, only mutations in SD4 completely abolished secretion of SseB, while causing enhanced secretion of SseJ. The derepressed secretion of SseJ shows that these mutants do not interfere with the essential function of SsaV in the export apparatus—for example, by destabilizing the nonameric ring structure. Rather, they are likely to reflect an altered interaction or loss of interaction between SsaV and the gatekeeper complex. This was verified with the SsaV^V632D^ mutant, which completely mimicked the secretion profile of the *ssaL* null mutant (Fig. 4) and no longer interacted with SsaL during growth at pH 5.0. We also found that the SctV homolog of the *Salmonella* SPI-1 T3SS, i.e., InvA, when mutated to InvA^V648D^, gave rise to a similar phenotype, mimicking that of the Δ*invE* gatekeeper mutant strain (16) with respect to both secretion profile and host cell invasion (Fig. 5).

Mutations of Shigella flexneri MxiA residues predicted to be involved in intersubunit salt bridges and hence in ring formation impair translocon protein secretion, but their effect on effector secretion was not reported previously (11). In another study, a MxiA^I674V^ mutant (located in SD4) had only a minor effect on secretion of translocon and effector proteins (42). A screen for *Pseudomonas* PcrD (SctV) mutants that derepress effector secretion yielded Q626R, which is located in SD4. While this mutant displayed very strong secretion of effectors, its effect on translocon protein secretion was not reported (41). Binding of immobilized peptides of the C-terminal region of E. coli EscV (SctV) to purified SepL (the E. coli homolog of SsaL) suggested that SepL binds to the external surface of EscV, forming a groove between two adjacent subunits (43). Mutation of V632 and V648 of SsaV and InvA phenocopied the secretion profile of the corresponding gatekeeper proteins (SsaL and InvE). Since the SVD (631 to 633) sequence is conserved in S. flexneri MxiA (Fig. 3), it would be interesting to determine if MxiA^V649D^ mimics the *mxiC* gatekeeper mutant in a similar way. The precise features and stoichiometry of the SctV-gatekeeper interface await purification and structural analysis of the bound complex, but our work strongly suggests that, at least for *Salmonella* SPI-2 T3SS, the gatekeeper is likely to dock within the inner lower clefts of the SsaV multimer and bind to surfaces of SD4.

How does the interaction between the gatekeeper and SctV enable the secretion of translocon proteins while preventing effector secretion? For SPI-1, it has been shown that InvE interacts with a complex formed by translocon protein SipB or SipC and their chaperone SicA (16). In the absence of InvE, the cytoplasmic sorting platform fails to recognize translocon proteins SipB, SipC, and SipD (12). It is therefore likely that a translocon-chaperone-InvE complex is loaded into the sorting platform via the interaction between InvE and InvA. A similar process might also occur in the T3SS of *Chlamydia* and *Shigella* (49). Binding of the gatekeeper in the clefts formed between SctV proteins could block effector-chaperone complexes from accessing the sorting platform, either by physical occlusion or through allosteric effects that prevent SctV from interacting with effector-chaperone complexes. Dissociation of SctV-gatekeeper interaction might then enable effector-chaperone complexes to access the sorting platform via the clefts between SctV subunits.

A dissociation of the gatekeeper-SctV complex might not be the only mechanism to trigger effector secretion. In the case of EPEC, purified SepL and the cytoplasmic region of EscV (EscV_C_) formed a complex in the presence or absence of calcium (44), suggesting that in bacteria, the SepL-EscV complex might not dissociate following sensing of a drop in calcium concentration. Instead, the conformation of the SepL-EscV complex might alter to enable effector-chaperone complexes to access the sorting platform via the clefts between EscV subunits. It was shown recently that EPEC ruler protein EscP interacts with SepL to regulate effector secretion (48). A Δ*escP* mutant mimicked the secretion profile of the gatekeeper mutant (33), and a EscP-SepL complex dissociated upon a drop in the calcium concentration to enable effector protein secretion (48). This mechanism might be specific to the T3SS of EPEC, as the ruler proteins from other T3SSs are essential for controlling the first secretion switch—from inner rod and needle subunits to translocon and effector proteins (28). In this study, we showed that SsaP and normal autocleavage of SsaU do not appear to be involved in the second secretion switch. However, they are both essential for secretion of translocon and effector proteins (Fig. 2A). Interestingly, we detected reduced interaction between SsaV and SsaL in the Δ*ssaP* mutant and the Δ*ssaU* mutant expressing SsaU^N262A^ (Fig. 6E), but this was not accompanied by secretion of effectors (Fig. 2C). This suggests that SsaP and SsaU are needed not only for the apparatus to become competent for secretion of translocon and effector proteins but also to control the SsaL-SsaV interaction temporally.

On the basis of our data presented here and elsewhere (18, 45–47), we propose a model for regulation of SPI-2 type III secretion as follows. (i) Low vacuole luminal pH levels are required to trigger SPI-2 gene expression and the production of SPI-2 proteins. (ii) Upon assembly of secretion apparatus, SsaG, SsaI, and SsaP are secreted, with SsaG forming the needle structure. In this state, the system is not competent for secretion of translocon or effector proteins. (iii) Once the needle reaches a certain length, SsaP and autocleaved SsaU cooperatively change the conformation of the secretion apparatus to enable the gatekeeper complex to interact efficiently with SsaV. (iv) The gatekeeper/SsaV complex delivers translocon proteins and their chaperones to the cytoplasmic sorting platform for secretion but prevents effectors from doing so. (v) After the translocon proteins form a pore in the vacuolar membrane, the neutral pH of the host cell cytosol is sensed by the secretion apparatus to dissociate the gatekeeper complex from the lower inner clefts of the SsaV ring, leading to degradation of SsaL/SpiC/SsaM. (vi) The newly exposed regions of the cytoplasmic domain of SsaV allow effectors and their chaperones to access the sorting platform for secretion and translocation.

Although all nf T3SSs appear to have a gatekeeper that controls the translocon-to-effector switch, a unified view of the underlying mechanism might not be possible, since each system appears to involve proteins with distinct characteristics. For example, the *Pseudomonas* and *Yersinia* tip protein chaperones PcrG and LcrG are involved in this process but are not found in other nf T3SSs (41, 50). For the SPI-2 system, future research topics include (i) the mechanism by which SsaU and SsaP function to enable translocon and effector secretion, (ii) the structure of the SsaV/gatekeeper complex, and (iii) how pH is sensed to cause its dissociation.

## MATERIALS AND METHODS

### Bacterial strains and growth conditions.

The *S.* Typhimurium strains used in this study are derivatives of wt strain 12023. SseJ-2HA expressed from chromosomal DNA in the wt strain, the Δ*ssaV*::*aphT* and Δ*ssaL*::*aphT* mutants was described previously (18, 51). Bacteria were grown in Luria-Bertani (LB) medium supplemented with carbenicillin (50 μg ml^−1^), kanamycin (50 μg ml^−1^), or chloramphenicol (15 μg ml^−1^), for strains resistant to these antibiotics (Carb^r^, Km^r^, and Cm^r^, respectively). To induce SPI-2 gene expression and SPI-2 T3SS-dependent secretion, bacteria were grown in MgM-MES at pH 5.0 (45) with the corresponding antibiotics where appropriate.

Using plasmid pKD4 as a template for PCR, the λ Red recombination system (52) was used to construct the following mutants: Δ*ssaP*::*Km* with primers ssaPd1 and ssaPd2 (primers are listed in Table 1), Δ*ssaU*::*Km* with primers ssaUd1 and ssaUd2, *ssaVΔ9*::*Km* with primers ssaVΔ9 and ssaVr-ch, *ssaV_neutral_*::*Km* with primers ssaV_neutral_ and ssaVr-ch, Δ*invA*::*Km* with primers invA600d1 and invA652d2, Δ*invE*::*Km* with primers invEd1 and invEd2, and *ssaV_Δ586-636_*::*Km* with primers ssaV586d1 and ssaV636d2.

**TABLE 1:** Primers used in this work

Name	Nucleotide sequence (5′ to 3′)
ssaPd1	TTACTATGGTATTAAGCGATGCGTATTACCAAAGTTGAGGGAGTGTAGGCTGGAGCTGCTTCG
ssaPd2	TCTTCATTCGCTATTCTTAACATAGAATATCTCCAGGGAAATCATATGAATATCCTCCTTAG
ssaGpf	ATCCTCGAGGTATGGATGGGATGGCAATGACC
ssaGpr	TCAAGCTTACACTAATTGTGCAATATCCATAATGC
ssaPf-HindIII	TCAAGCTTACAGAAGAATTTTAATGCGC
ssaPr-SacI	ATCGAGCTCCACCCACGGACGCTCTTCATTCGC
ssaUd1	AAAAGACTGGTTTCCATCTGTATGAGCGAGAAAACAGAACGTGTAGGCTGGAGCTGCTTC
ssaUd2	TTAACCTTCGCAGTGGCCTGAAGAAGCATACCAAAAGCATATGAATATCCTCCTTAGT
ssaUf-EcoRV	TCGATATCAAAAGACTGGTTTCCATCTG
ssaUr-SacI	CAGAGCTCTTAGTGGTGGTGGTGGTGGTGTGGTGTTTCGGTAGAATGCGC
ssaU^N262A^f	GTTGCGGTAGTGCGTGCTCCAACGCATATTGCG
ssaU^N262A^r	CGCAATATGCGTTGGAGCACGCACTACCGCAACAG
ssaVf-HindIII	TCAAGCTTCCAGCTCCGCCGAGCTCTGG
ssaVr-PstI	AACTGCAGAATTCATTCTTCATTGTCCGCC
ssaV672r-PstI	AACTGCAGTCAGCTAAGGTCAATACTTTCTACC
ssaVΔ9	GAGGAGAGCCTTATACAAGTGGTAGAAAGTATTGACCTTAGCTAGGTGTAGGCTGGAGCTGCTTC
ssaV_neutral_	GAGGAGAGCCTTATACAAGTGGTAGAAAGTATTGACCTTAGCCAACAGCAGTTGGCGAACAATCAACAATAGGTGTAGGCTGGAGCTGCTTC
ssaVr-ch	TCGGGGGGCGGATATTTCAGCCTCAGACGTTGCATCAATTCATTCTTCATCATATGAATATCCTCCTTAGT
ssaV586d1	GGAAGGAAAACCGCTGCCGATTTTGCGGATCGGCGAAGGTATTGAGTGTAG GCTGGAGCTGCTTC
ssaV636d2	AATCGGTACGTCGAACAAGGTGGCTTCTGTAATTTTTCGCAAGAACATATGAATATCCTCCTTAGT
ssaV541f-SphI	TTAGCATGCATTGACTGGGCGCCACGTG
ssaVr-EcoRI	CGGAATTCTCATTCTTCATTGTCCGCCAAC
invA600d1	ATGGCGGCGAATTACGAGCAGTAATGGTATCTGCTGAAGTTGAGGATGTGTA GGCTGGAGCTGCTTC
invA652d2	AACCTCCAGATCCGGAAAACGACCTTCAATCATTTTCTTAATAAACATATGAA TATCCTCCTTAGT
invA561f-SphI	TTAGCATGCAAAGATGTCATTAACCTTGTGGAGC
invAr-EcoRI	CGGAATTCTTATATTGTTTTTATAACATTCACTGACTTGC
invA^V648D^f	TCCTCCTTACGTCTGACGATGTCCGTCGATTTATTAAG
invA^V648D^r	AAATCGACGGACATCGTCAGACGTAAGGAGGACAAGATC
invEd1	GACATCTCATCAGGATGCGACCCAGCATACTGATGCGCAACAGGCGGGTGTAGGCTGGAGCTGCTTC
invEd2	GCAATATCGGTCATACTACGTAACGCCATTAACAATTCTTCCTGCCCATATGAATATCCTCCTTAGT
ssaLd1	GCTATAGTTTCTTCATCGAAGATGTTCAATCGGTTACTCCAACAAGTGTAGGCTGGAGCTGCTTC
ssaMd2	TTTCATATTGTCCTTGCCGCCAGAACATATTGGTTGCTAAAGGCATATGAATATCCTCCTTAGT
ssaL’f-SphI	TTAGCATGCCGGGTCCGTATTTTGCTAAGAGCAGTAGC
ssaL3flagr	CTTTGTAGTCGATATCATGATCTTTATAATCACCGTCATGGTCTTTGTAGTCGAATAAAACCTGATTTATCTTTACTTCACG
ssaL3flagf	ATGACGGTGATTATAAAGATCATGATATCGACTACAAAGATGACGACGATAAATAAATCAGGTTTTATTCTGATACCTGGCTTTC
ssaMr-EcoRI	CGGAATTCCTAACCATGAACGCATTGCGACTCC
ssaVhis	AGTATTGACCTTAGCGAAGAGGAGTTGGCGGACAATGAAGAACACCACCATCATCACCATTAG

Chromosomal allelic exchange was used to construct the *ssaV^V632D^* and *invA^V648D^* mutants. For *ssaV^V632D^*, primers ssaV541f-SphI and ssaVr-EcoRI were used to amplify DNA encoding residues 541 to 681 of SsaV^V632D^ from plasmid p*ssaV^Q251L,V632D^* (obtained from random-mutagenesis screening). The PCR product was digested and ligated into the SphI and EcoRI sites of suicide vector pGP704 (53) to create plasmid pGP-ssaVc^V632D^. pGP-ssaVc^V632D^ was transferred into the *ssaV_Δ586-636_*::*Km* mutant by conjugation, and exconjugants were selected as previously described (54). To make the *invA^V648D^* mutant, overlapping PCR was carried out to obtain the DNA sequence encoding residues 561 to 685 of InvA^V648D^ (a first-round PCR was done using *Salmonella* genomic DNA as the template with primers invA561f-SphI and invAV648Dr and primers invAV648Df and invAr-EcoRI, respectively; the second-round PCR was done by using a mixture of first-round PCR products as the template with primers invA561f-SphI and invAr-EcoRI). The second-round PCR product was digested and ligated into the SphI and EcoRI sites of pGP704 to create plasmid pGP-invAc^V648D^. pGP-invAc^V648D^ was transferred into the Δ*invA*::*Km* mutant by conjugation, and exconjugants were selected.

To tag *ssaL* with 3Flag, the DNA sequence containing *ssaL-3Flag* and *ssaM* was amplified via overlapping PCR by using primers ssaL’f-SphI and ssaL3Flagr and primers ssaL3Flagf and ssaMr-EcoRI, respectively, for the first-round PCR and primers ssaL’f-SphI and ssaMr-EcoRI for the second-round PCR. The second-round PCR product was digested and ligated into the SphI and EcoRI sites of pGP704 to create plasmid pGP-ssaL3Flag/ssaM. Plasmid pGP-ssaL3Flag/ssaM was transferred into the Δ*ssaLc* Δ*ssaMn*::*Km* mutant (created by the λ Red recombination system with primers ssaLd1 and ssaMd2) by conjugation, and exconjugants were selected to make strain *ssaL-3Flag*. To create strain *ssaL-3FlagssaV^V632D^*, the *ssaV^V632D^* mutation was introduced into the *ssaL-3Flag* strain using a technique similar to that used to construct the *ssaV^V632D^* mutant. The PCR product obtained by using pSUB7 (55) as the template and oligonucleotides ssaVhis and ssaVr-ch as primers was transferred into the *ssaL-3Flag* strain or the *ssaL-3FlagssaV^V632D^* strain carrying pKD46 to create the *ssaL-3Flag*/*ssaV-6his*::*Km* strain or *ssaL-3FlagssaV^V632D^*-6his::Km strain.

When necessary, the FRT-flanked antibiotic resistance cassette in strains made by the λ Red recombination system was removed after transformation with pCP20, as described previously (52). The Δ*ssaP*::*Km* and Δ*ssaU*::*Km* mutations were transduced into different *S.* Typhimurium strains by the use of phage P22 (56).

### Plasmids.

The DNA sequence of the *ssaG* promoter was amplified from *Salmonella* genomic DNA with primers ssaGpf and ssaGpr, digested with XhoI and HindIII, and ligated into the XhoI and HindIII sites of pWSK29 (57) to create p*ssaG_pr_*. Plasmid p*ssaP* was constructed by ligating a HindIII- and SacI-digested PCR product of *ssaP* (amplified with primers ssaPf-HindIII and ssaPr-SacI) into the same sites of p*ssaG_pr_*.

The 6×His-tagged *ssaU* sequence was amplified with primers ssaUf-EcoRV and ssaUr-SacI, digested with EcoRV and SacI, and ligated into the same sites of p*spiC_pr_* (58) to create plasmid p*ssaU-6his*. To construct plasmid p*ssaU^N262A^-6his*, the first-round PCR products with primers ssaUf-EcoRV and ssaU^N262A^r and primers ssaUr-SacI and ssaU^N262A^f were recovered from an agarose gel to serve as a template for a second-round PCR with primers ssaUf-EcoRV and ssaUr-SacI. The second-round PCR product was digested with EcoRV and SacI and was ligated into the same sites of p*spiC_pr_* to create plasmid p*ssaU^N262A^-6his*.

Plasmids p*ssaV* and p*ssaVΔ9* were constructed by ligating HindIII- and PstI-digested PCR products of ssaV (amplified with primers ssaVf-HindIII and ssaVr-PstI) or ssaV*Δ9* (amplified with primers ssaVf-HindIII and ssaV672r-PstI) into the same sites of p*ssaG_pr_*.

All the plasmids constructed in this study were verified by DNA sequencing. Plasmid p*sseF-2HA* expressing SseF-2HA under the control of the SPI-2 promoter and plasmid pM1301 expressing SipA-M45 under the control of the SPI-1 promoter were described previously (18, 59).

### Antibodies.

The following primary antibodies were used for immunoblot analysis: rabbit anti-SseB (1:10,000), anti-SseC (1:10,000), and anti-SseD (1:10,000) (51); rabbit anti-SsaI (1:5,000), anti-SipB (1:25,000), and anti-SipC (1:20,000) (provided by Akiko Takaya, Chiba University, Japan); rabbit anti-SopE2 (1:5,000) and mouse anti-M45 (1:2,500) (provided by Wolf-Dietrich Hardt, Institute of Microbiology, ETH Zurich, Switzerland); mouse anti-hemagglutinin (anti-HA) (MMS-101P; Covance) (1:5,000); mouse anti-DnaK (Assay Designs) (1:10,000); and mouse anti-Flag M2 (Sigma) (1:2,000).

Horseradish peroxidase (HRP)-conjugated goat anti-mouse Igs and anti-rabbit Igs (Dako) were used at a dilution of 1:5,000. HRP-conjugated mouse anti-His (Abcam) was used at a dilution of 1:2,500.

### Random mutagenesis of *ssaV* to screen for enhanced secretion of SseJ.

Plasmid *pssaV* was used to randomly mutagenize the *ssaV* gene by using a GeneMorph II EZClone domain mutagenesis kit (Agilent Technologies) together with primers ssaVf-HindIII and ssaVr-PstI. Following transformation into E. coli, sequencing of 10 individual plasmids revealed a range of mutations with no duplications. The pool of plasmids isolated from E. coli was transformed into the Δ*ssaV*::*aphT* mutant expressing SseJ-2HA from the chromosome and plated onto LB-plus-Carb medium. Individual transformants were patched onto solid MgM-MES at pH 5.0 containing carbenicillin and grown overnight at 37°C. Then, a nitrocellulose membrane (WhatmanProtran BA85; GE Healthcare) (0.45-µm pore size) was placed onto the surface of colonies and the colonies were incubated for another 7 h at 37°C to allow secreted proteins to be transferred onto the membrane. The membrane was then gently lifted up and put into a tube containing phosphate-buffered saline (PBS) with 0.2% Tween 20 and 5% semiskimmed milk powder. The tube was kept on a roller at 4°C overnight. The membrane was washed three times with PBS–0.1% Tween 20, incubated with a mouse anti-HA antibody followed by HRP-conjugated goat anti-mouse Ig antibody, and examined using an enhanced chemiluminescence (ECL) detection system.

SseJ-2HA-positive colonies were subjected to a secretion assay by Western blotting to verify enhanced secretion of SseJ-2HA and to check secretion of SseB. Plasmids were isolated from strains showing enhanced secretion of SseJ-2HA and were sequenced to determine the mutation(s) in *ssaV*.

### Secretion and coimmunoprecipitation assays.

For the SPI-2 secretion assay done at pH 5.0, an overnight bacterial culture in LB was subcultured into MgM-MES at pH 5.0 at a dilution of 1:50 and was grown for 6 h in a 37°C shaker. For pH shift analysis, the subculture was grown for 4 h at pH 5.0 and switched after centrifugation (at 7,000 × *g*) into new MgM-MES at pH 5.0 or 7.2 for another 1.5 h. The whole-bacterium lysate and secreted fraction were prepared as described previously (51) to make 10 µl of whole-bacterium lysate equal to an optical density at 600 nm (OD_600_) of 0.1 of culture and 10 µl of secreted fraction equal to an OD_600_ of 0.6 of culture.

For the SPI-1 secretion assay, an overnight bacterial culture in LB was subcultured into LB at a dilution of 1:33 and grown for 3.5 h in a 37°C shaker. The bacterial culture was centrifuged at 12,000 × *g*, and the supernatant was filtered through a 0.45-µm-pore-size membrane and precipitated with 10% trichloroacetic acid. To ensure that protein was analyzed from equal numbers of bacteria, in all experiments, protein samples were adjusted to OD_600_ values in protein loading buffer to make 10 µl of whole-bacterium lysate equal to an OD_600_ of 0.1 of culture and 10 µl of supernatant equal to an OD_600_ of 0.16 of culture.

For coimmunoprecipitation assay, bacterial strains were grown for 6 h in 10 ml of MgM-MES at pH 5.0 before collection by centrifugation. For the pH shift experiment, bacterial strains were grown for 4 h in 12 ml of MgM-MES at pH 5.0 and then resuspended into 10 ml of MgM-MES at pH 7.2 to grow for another 2 h before collection. The bacterial pellet was resuspended into 950 µl of PBS–1mM phenylmethylsulfonyl fluoride (PMSF) for ultrasonication (Bandelin Sonopuls untrasonic homogenizer) (2 min with MS72 probe at 50% power), and then 50 µl of 10% Triton X-100 was added and the reaction mixture was incubated at 4°C for 1 h. Following centrifugation at 16,000 × g for 5 min, the supernatant was precleaned with 40 µl of protein G beads for 1 h. A 40 µl volume of anti-Flag M2 affinity agarose gel (Sigma) were mixed with precleaned lysate and incubated for 3 h at 4°C. The immunoprecipitates were washed four times with PBS–1mM PMSF–0.1% Triton X-100 and twice with PBS and were then eluted with 50 µl of 0.3 mg/ml 3Flag peptide.

Protein samples were separated through 12% SDS-polyacrylamide gels and transferred to Immobilon-P polyvinylidene difluoride (PVDF) membranes (Merck) for immunoblotting. The intrabacterial protein DnaK was used as a control for the secreted fractions. All Western blotting data are representative of results of at least three independent experiments.

### Invasion and replication analysis.

HeLa cells (clone HtTA1) and RAW264.7 cells (ECACC 91062702) were grown in Dulbecco’s modified Eagle’s medium (DMEM)-high glucose (Sigma) supplemented with 10% fetal calf serum at 37°C in 5% CO_2_.

For invasion assays, HeLa cells were infected with exponential-phase *S.* Typhimurium as described previously (60). At 2 h postchallenge, cells were lysed with PBS containing 0.1% Triton X-100, and the lysate was plated onto LB medium to count the number of intracellular bacteria. The invasion rate was calculated as the ratio between the number of intracellular bacteria and the number of bacteria used for infection.

For intracellular replication assays, RAW264.7 cells were infected with stationary-phase *S.* Typhimurium, and the fold increase was determined as the ratio between the number of intracellular bacteria at 16 h postuptake and the number of intracellular bacteria at 2 h postuptake (60).

Both the invasion and the replication assays were done three times and each time in triplicate.

### Bioinformatic analysis and modeling of SsaV.

The alignment of SsaV and its homologues was generated by Clustal Omega (https://www.ebi.ac.uk/Tools/msa/clustalo/). The cytosolic region of SsaV (SsaV^342–672^) was modeled from MxiA^318–686^ via Phyre2 (http://www.sbg.bio.ic.ac.uk/phyre2/html/page.cgi?id=index) and displayed using Pymol software.

### Statistical analysis.

Statistical analysis was carried out with Prism 5 software (GraphPad) using one-way analysis of variance (ANOVA) and Dunnett’s *post hoc* analysis. Probability (*P*) values of 0.05 or less were considered significant.
